# An Interesting Outcome of Central Sleep Apnea With Phrenic Nerve Stimulation Therapy

**DOI:** 10.7759/cureus.92603

**Published:** 2025-09-18

**Authors:** Seshadri Jagannathan, Ram Verma

**Affiliations:** 1 Internal Medicine, Parkview Regional Medical Center, Fort Wayne, USA; 2 Sleep Medicine, Parkview Regional Medical Center, Fort Wayne, USA

**Keywords:** central sleep apnea, cognition, hypoxia, mathematical skills, remede device, stroke, verbal fluency

## Abstract

Central sleep apnea (CSA) is secondary to inadequate ventilation during sleep, causing individuals to experience repeated hypoxic episodes with long-term consequences of oxygen desaturation, such as arrhythmias, hypertension, congestive heart failure, stroke, coronary artery disease, cognitive issues, increased risk of accidents, type 2 diabetes, and worsening Parkinson’s disease. The phrenic nerve stimulator, remedē System (ZOLL Respicardia, Inc., Minnetonka, MN) is indicated as treatment for moderate-to-severe CSA in adults. It works by stimulating the phrenic nerve, which, in turn, causes the diaphragm to contract and helps restore normal breathing patterns during sleep. A 60-year-old male with obesity, essential hypertension, seizure, and stroke with biventricular hemorrhage secondary to cerebral aneurysm was seen in a sleep medicine clinic for sleep apnea. His polysomnogram showed severe primary CSA with an apnea-hypopnea index (AHI) of 63.4 per hour, central apnea index of 51.8 per hour, and lowest oxygen saturation of 73% with nocturnal hypoxemia of 6.9 minutes below 88% oxygen saturation. After remedē System implantation, the patient’s WatchPAT® sleep study showed a residual AHI of 11.4 per hour, CSA index of 0.0 per hour, and lowest oxygen saturation 84% with nocturnal hypoxemia of 3.2 minutes below 88% oxygen saturation. In our unique case, we found significant improvement of AHI and resolution of CSA along with improvement in verbal fluency, mathematical skills, and cognition with the use of remedē System.

## Introduction

Stroke can significantly impact cognitive function, leading to a range of challenges with thinking, memory, and problem-solving. There are two main types of stroke: ischemic and hemorrhagic. Ischemic strokes are caused by a blockage in a blood vessel, while hemorrhagic strokes are caused by bleeding in the brain. This case report is unique as treatment of central sleep apnea (CSA) with phrenic nerve stimulation (PNS) therapy improved sleep, cognition, and quality of life.

Sleep apnea, particularly obstructive sleep apnea (OSA), is common after a stroke, with prevalence rates significantly higher than in the general population. CSA can also occur after stroke, though less frequently than OSA. There are several types of CSA, including high altitude-induced periodic breathing, idiopathic CSA, narcotic-induced central apnea, obesity hypoventilation syndrome, and Cheyne-Stokes breathing [1]. Sleep-disordered breathing (SDB) increases the risk of hypertension, coronary artery disease, heart failure, and atrial fibrillation [2].

CSA can be idiopathic or occur along with Cheyne-Stokes respiration (abnormal breathing with alternate rapid and shallow breathing), chronic kidney disease, or high-altitude periodic breathing. Risk factors include sex, age, congestive heart failure, opioid use, cerebrovascular accidents, and other chronic medical issues [3].

SDB, such as OSA and CSA, is an independent risk factor for cerebrovascular accidents, congestive heart failure, pathological heart rhythms, and other cardiovascular conditions. The effects of OSA on brain structure and cognitive function have a potential role in the development of neurocognitive abnormalities [4].

Clinically, CSA is noted in association with OSA, heart failure, atrial fibrillation, and chronic opioid use [5]. It increases morbidity and mortality in congestive heart failure patients due to the repeated hypoxic episodes and norepinephrine release [6]. Traditional treatment of CSA includes continuous positive airway pressure (CPAP) therapy, machines such as adaptive servo ventilation (ASV), oxygen therapy, and carbon dioxide inhalation [7].

The phrenic nerve stimulator remedē System (ZOLL Respicardia, Inc., Minnetonka, MN) is indicated as treatment for moderate-to-severe CSA in adults. It works by stimulating the phrenic nerve, which, in turn, causes the diaphragm to contract and helps restore normal breathing patterns during sleep. Transvenous PNS is a new method available for the treatment of CSA [8]. Though the remedē System device is closer to the heart structures on X-rays or other imaging, it does not have any pacemaker or defibrillator abilities, and it is important to recognize and manage such a device if they were to come across prior to surgical interventions [8]. CSA patients often have other medical comorbidities that may benefit from MRI scanning, including, but not limited to, brain, spine, and joint issues. Approval of the remedē System for conditional use with MRI will allow patients to have access to this important diagnostic imaging tool [9].

There is available information suggesting that sleep apnea causes cognitive impairment. Data suggest that verbal fluency impairment is associated with inadequate non-rapid eye movement (NREM) sleep [10] and that OSA is associated with cognitive impairment more than CSA [10].

Hypoxemic burden has been demonstrated to be more predictive for mortality than apnea-hypopnea index (AHI). It should be considered while treating CSA, and PNS has shown to improve nocturnal hypoxemic burden [11,12].

## Case presentation

A 60-year-old male presented with stroke, excess weight, essential hypertension, and post-stroke seizure with biventricular hemorrhage secondary to rupture of cerebral aneurysm. He was treated with a ventricular drain. His post-stroke seizure was treated with lamotrigine. He was seen in a sleep medicine clinic around one year after his stroke for the evaluation of sleep apnea due to excessive daytime sleepiness. His vitals were stable in the sleep clinic. The patient was found to have excessive daytime sleepiness, lethargy, and impaired cognitive function. His polysomnogram showed severe primary CSA, with an overall AHI of 63.4 per hour, central apnea index of 51.8 per hour, and lowest oxygen saturation of 73% with nocturnal hypoxemia of 6.9 minutes below 88% oxygen saturation. He underwent titration study, where he was found to have insufficient treatment response with both CPAP and BiPAP (bilevel positive airway pressure). The patient was recommended ASV, but unfortunately he did not tolerate ASV due to his cognitive impairment following the stroke.

The patient underwent PNS therapy (remedē) implantation with activation around one year after initial sleep study due to ASV intolerance. After one year of implantation, his WatchPAT® sleep study showed residual AHI of 11.4 per hour, CSA index of 0.0 per hour, and lowest oxygen saturation of 84% with nocturnal hypoxemia 3.2 minutes below 88% oxygen saturation. His verbal and mathematical skills improved since remedē device activation. The patient was lethargic prior to remedē device implantation and needed stimulant medications to stay awake. His verbal fluency improved. His mathematical skills were stagnant at the kindergarten level prior to remedē implantation. The patient was recently seen around four years post-remedē implantation and was found to have improved verbal and mathematical skills to the fifth-grade level. Overall, the patient showed great improvement in cognition and other skills of daily living with the remedē device implantation.

He is able to help his wife now with daily chores at home, loading the dishwasher, and other work. The patient still has some fear of taking a shower himself, otherwise he is doing much better in other aspects. Overall, his recovery of stroke is much better with the remedē device implantation.

## Discussion

Hypoxia, or a deficiency of oxygen in the brain, can significantly impair cognitive function. Both acute and chronic hypoxia can lead to impairments in various cognitive domains, including attention, memory, processing speed, and executive function [13]. Moreover, brain structural changes with hippocampus and cortex atrophy, ventricle enlargement, senile plaque, and neurofibrillary tangle deposition can be observed under chronic hypoxia rather than acute hypoxia [13].

OSA with repetitive intermittent apnea and hypoxia causes neuronal injury by inflammation in areas of the hippocampus and cortex, leading to cognitive dysfunction [14]. It is known that CPAP treatment can help alleviate intermittent hypoxia-induced neurocognitive dysfunction [14]. There is limited research literature on CSA causing cognitive deficits. A large five-year prospective study is ongoing regarding the safety and effectiveness of the remedē system as part of the treatment for moderate-to-severe CSA in adult patients [15]. However, there is another study showing improvement in sleep parameters, sleep symptoms, and quality of life with the use of transvenous PNS over one year of follow-up [16].

In our case report, we found significant improvement of AHI with the resolution of CSA and hypoxemia using PNS therapy device. This case report is unique as it focused on the outcome of his verbal and mathematical skills and the ability to function, with improvement in activities of daily living after the remedē system implantation. While the remedē System does not directly impact cognition, improving sleep through its treatment can indirectly benefit cognitive function by reducing daytime sleepiness and improving overall well-being.

## Conclusions

We should screen and treat stroke patients for both OSA and CSA, which may help in stroke recovery. Unfortunately, some patients with SDB are intolerant to non-invasive positive airway pressure (PAP) therapy because of various factors such as physical limitations from the stroke, anxiety, and difficulty in mask fit. Intolerance to PAP can lead to reduced adherence, potentially impacting their recovery from stroke and increasing the risk of cardiovascular events. Improvement of sleep with PNS device therapy in CSA patients leads to improved cognition in verbal and mathematical skills as well as improvement in activities of daily living.
